# One-year outcome of transcatheter aortic valve replacement for bicuspid aortic valve stenosis: a report from the Japanese Nationwide registry (J-TVT registry)

**DOI:** 10.1007/s12928-023-00933-y

**Published:** 2023-06-06

**Authors:** Takashi Mukai, Hiraku Kumamaru, Shun Kohsaka, Isamu Mizote, Daisuke Nakamura, Yutaka Matsuhiro, Koichi Maeda, Kazuo Shimamura, Yasushi Sakata

**Affiliations:** 1 Department of Cardiovascular Medicine, Osaka University Graduate School of Medicine, 2-2 Yamadaoka, Suita, Osaka 565-0871 Japan; 2 Department of Healthcare Quality Assessment, Graduate School of Medicine, The University of Tokyo, Tokyo, Japan; 3 Department of Cardiology, Keio University, Tokyo, Japan; 4 Department of Cardiovascular Surgery, Osaka University Graduate School of Medicine, Osaka, Japan

**Keywords:** Aortic valve stenosis, Bicuspid aortic valve, Transcatheter aortic valve replacement

## Abstract

The outcome of transcatheter aortic valve replacement (TAVR) for patients with bicuspid aortic valve (BAV) remains unclear, particularly among Asian patients that are known to have different valvular morphology and lower body habitus. This study investigated patient characteristics, procedural and 1-year outcome of TAVR for BAV within national TAVR registry in Japan. The patient‐level data were extracted from the J-TVT (Japanese Transcatheter Valvular Therapy) registr*y* between August 2013 and December 2018; overall, there were 423 patients (2.5%) with BAV and 16,802 patients with tricuspid aortic valve (TAV). At baseline, patients with BAV were younger and had less arteriosclerotic comorbidities. There was no statistically significant difference between BAV and TAV groups in conversion to surgery (0.5% vs. 1.1%, *p = *0.34) and 30-day mortality (0.5% vs. 1.3%, *p = *0.18). Cumulative all-cause survival and survival from major adverse events were analyzed. Cox proportional hazard regression model was used to estimate the hazard ratio. All-cause mortality and major adverse event rate at 1 year were comparable between the two groups. Relative hazard for all-cause mortality for BAV compared to TAV was 1.01 (0.70–1.45; *p = *0.96), and for major adverse event was 0.94 (0.69–1.27; *p = *0.67). From the Japanese nationwide TAVR registry, procedural and 1-year outcome of TAVR in BAV was as favorable as TAVR in TAV.

## Introduction

Bicuspid aortic valve (BAV) is one of the most frequently encountered congenital cardiac disorder [1], and on the basis of surgical experience, up to 50% of younger patients requiring aortic valve intervention have a BAV morphology. In the contemporary era, increasing number of patients presenting with BAV are considered as a candidate for transcatheter aortic valve replacement (TAVR) as the age of the patients being treated decreases. Typically, BAV are associated with anatomical features that make TAVR more challenging. BAV patients often present asymmetrical cusps, bulky asymmetrical calcification or commissural fusion, and are thought to be at higher risk for aortic root rupture and paravalvular leakage compared to patients with tricuspid aortic valve (TAV) [2, 3].

To date, patients with BAV have been excluded from the pivotal randomized trials on TAVR. Recent observational studies have demonstrated favorable short- or mid-term outcome of TAVR in BAV [4], and this has been replicate in patients who are younger and at low-risk [5, 6]. This is of clinical significance as BAV is more frequently encountered in patients undergoing SAVR who are aged 70–79 years than in those aged 80–89 years. Moreover, the proportion of BAV varies substantially across the regions. There are inter-ethnic differences in BAV morphology between Asian and European patients; BAVs with no raphes type were less prevalent among Asian patients than European patients [7]. Further, smaller body size of Asian population may be associated with higher risk of annulus rupture and vascular complications due to their smaller aortic annulus size and access site vascular diameter [8]. These differences may have important implication in assessment of the safety and efficacy of TAVR among Asian patients.

In the present study, we analyzed the contemporary Japanese nationwide database and evaluated the clinical outcome of TAVR in BAV patients. The Japanese TAVR registry (Japan Transcatheter Valve Therapies [J-TVT] registry) was launched in 2013. J-TVT registry institutions and operators are mandated to consecutively register all cases at baseline and their 1-year follow-up. With over 18,000 consecutive patients registered to date, the J-TVT registry provides a unique opportunity to elucidate the performance of TAVR in BAV in Japan.

## Methods

### Database

The J-TVT registry is endorsed by 4 Japanese academic societies (The Japanese Circulation Society, the Society of Japanese Cardiovascular Surgery, the Japanese Association of Thoracic Surgery, and the Japanese Association of Cardiovascular Intervention and Therapeutics) to develop a database about TAVR procedures in Japan. Details on J-TVT registry has been described previously [9]. Registration of all TAVR cases is one of the essential requirements for the certification of the institutions and operators for TAVR. At each participating site, the institutional review board or director of the institution approved the registration of patient information into the database with an opt-out process according to the ethical human subject guidelines, published by the Ministry of Education, Culture, Sports, Science and Technology, and the Ministry of Health, Labor and Welfare of Japan (2005). The current study was approved by Osaka University Hospital institutional ethical review board.

### Patient selection and study design

**Fig. 1:**
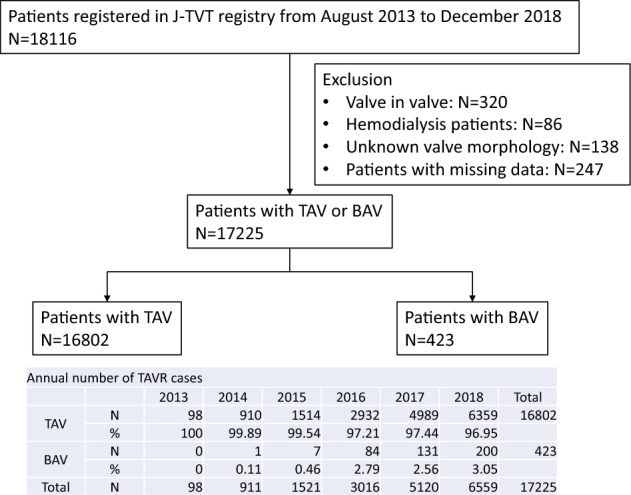
Flow of the patient selection

We analyzed patients registered in the J-TVT registry that underwent TAVR between August 2013 and December 2018 in Japan. Data on the nature of the aortic valve were retrieved solely from the procedural report of patients. A total of 18,116 patients were treated with TAVR in Japan between August 2013 and December 2018 in Japan. We excluded patients with valve-in-valve procedure (*n = *320, 1.8%) and patients without information on valve morphology (*n = *138, 0.8%). Patients with hemodialysis (*n = *86, 0.5%) or lacked follow-up data (*n = *247, 1.4%) were also excluded from the analysis. Consequently, 423 patients with BAV (2.5%) and 16,802 patients with TAV (97.5%) were included. Patient selection flow is presented in Fig. 1. Clinical outcomes of TAVR were compared between BAV group and TAV group.

### Outcomes

Transcatheter aortic valve replacement-specific outcomes were defined according to the Valve Academic Research Consortium 3 (VARC-3) [10]. The primary outcome of interests were overall survival and survival from major adverse events. We defined major adverse events as a composite of severe adverse events (major bleeding, stroke, myocardial infarction, and heart failure hospitalization) and death from any cause.

### Statistical analysis

We compared the baseline patient characteristics between BAV group and TAV group. We also compared the incidences of periprocedural outcomes between the two groups and tested the difference, using the chi-squared test or Fisher’s exact test as appropriate for categorical factors, and the Wilcoxon rank sum test for continuous variables. Continuous variables are expressed as medians with the interquartile range (IQR). Cumulative all cause survival and survival from major adverse events were analyzed using the Kaplan–Meier method, and were compared using log-rank test. Patients were followed from the day of the procedure until the earlier of their last date of survival reported in the registry or 365 days post procedure. We also constructed Cox proportional hazard models for the outcomes described above with and without including the following clinical characteristics in the model: age, sex, body surface area (BSA), New York heart association (NYHA) III/IV, Society of Thoracic Surgeons (STS) score, comorbidities including hypertension, hyperlipidemia, diabetes mellitus, chronic lung disease, coronary artery disease, non-cardiac artery disease, prior percutaneous coronary intervention (PCI), prior coronary artery bypass grafting, prior heart surgery, cerebrovascular disease, pacemaker implantation, aortic calcification and malignancy, laboratory data of hemoglobin, albumin and creatinine, echocardiographic data including mean aortic valve gradient, aortic valve area, left ventricular ejection fraction (LVEF), aortic insufficiency 3–4 and mitral insufficiency 3–4, drug use of warfarin, aspirin, ticlopidine and clopidogrel or prasugrel were included to compare the hazard for patients with BAV and TAV. Among the explanatory variables, age, BSA, STS score, hemoglobin, albumin, creatinine, mean aortic valve gradient, aortic valve area, LVEF were included as continuous variables, and the others were binary variables.

## Results

The number of BAV patients that underwent TAVR has gradually increased year by year (Fig. 1). The proportion of BAV was 2.5%. The prevalence of BAV in TAVR was reported as 2–6% previously [11, 12]. Some recent study analyzing STS/ACC TVT registry database reported that the proportion of BAV was 3.3–3.5% [13, 14]. This is slightly large proportion, but not so far from the proportion of BAV (2.5%) in J-TVT registry. One possible reason to explain the difference is that SAVR might be chosen more frequently in BAV patients than TAV patients if they had the same risk of surgery, because safety and efficacy of TAVR in BAV patients were unclear during the study period in Japan. Clinical baseline characteristics are listed in Table 1. Patients with BAV were younger and more likely to be male, compared to TAV group. Hypertension, hyperlipidemia, coronary artery disease and prior percutaneous coronary intervention was less frequent in BAV group. Society of Thoracic Surgeons (STS) score was lower in BAV group (5.4% [IQR: 3.5–8.2] vs. 6.1% [IQR: 4.4–8.7], *p < *0.001). Preoperative echocardiography showed higher mean pressure gradient of aortic valve in BAV group (54.0 mmHg [IQR: 41.2–67.0] vs. 47 mmHg [IQR: 38.0–60.0], *p < *0.001), lower ejection fraction (62% [IQR: 51–68] vs. 64% [IQR: 56–70], *p < *0.001), higher prevalence of moderate or severe aortic insufficiency (11.8% vs. 7.5%, *p < *0.001).

**Table 1:** Baseline characteristics

	TAV group	BAV group	*p* value
N	16,802	423	
Age			
< 65 years	41 (0.2)	8 (1.9)	< 0.001
< 75 years	586 (3.5)	50 (11.8)	
< 85 years	7297(43.4)	201 (47.5)	
85 years and above	8849 (52.7)	164 (38.8)	
Missing	29 (0.2)	0 (0.0)	
Male	5085 (30.3)	225 (53.2)	< 0.001
BSA	1.41 (1.30–1.54)	1.48 (1.34–1.61)	< 0.001
NYHA 3–4	4286 (25.5)	115 (27.2)	0.45
STS score	6.1 (4.4–8.7)	5.4 (3.5–8.2)	< 0.001
Comorbidities			
Hypertension	13,335 (79.4)	278 (65.7)	< 0.001
Hyperlipidemia	8060 (48.0)	159 (37.6)	< 0.001
Diabetes mellitus	4429 (26.4)	90 (21.3)	0.02
Chronic lung disease	1367 (8.1)	46 (10.9)	0.043
Coronary artery disease	5412 (32.2)	89 (21.0)	< 0.001
Non cardiac artery disease	2171 (12.9)	43 (10.2)	0.09
Prior PCI	3990 (23.7)	52 (12.3)	< 0.001
Prior CABG	855 (5.1)	13 (3.1)	0.06
Prior heart surgery	1432 (8.5)	31 (7.3)	0.38
Cerebrovascular disease	2011 (12.0)	57 (13.5)	0.35
Pacemaker	982 (5.8)	22 (5.2)	0.58
Aortic calcification	1597 (9.5)	33 (7.8)	0.23
Malignancy	1498 (8.9)	62 (14.7)	< 0.001
Labo data			
Hemoglobin	11.3 (10.2–12.4)	12.0 (10.8–13.1)	< 0.001
Albumin	3.8 (3.4–4.1)	3.8 (3.4–4.1)	0.21
Creatinine	0.9 (0.7–1.2)	0.9 (0.7–1.2)	0.17
Echocardiographic data			
Peak Aortic Valve Gradient	79.0 (65.0–100.0)	89.0 (71.8–111.6)	< 0.001
Mean gradient	47.0 (38.0–60.0)	54.0 (41.2–67.0)	< 0.001
AV area	0.6 (0.5–0.7)	0.6 (0.5–0.7)	0.053
LVEF	64 (56–70)	62 (51–68)	< 0.001
Aortic insufficiency grade 3–4	1252 (7.5)	50 (11.8)	< 0.001
Mitral insufficiency grade 3–4	1340 (8.0)	39 (9.2)	0.36
Drug use			
Warfarin	1045 (6.2)	23 (5.4)	0.51
Aspirin	7924 (47.2)	163 (38.5)	< 0.001
Clopidogrel or Prasugrel	4256 (25.3)	75 (17.7)	< 0.001
Ticlopidine	124 (0.7)	3 (0.7)	1.00

Chronic lung disease is defined by a forced expiratory volume over one second (FEV1) less than 60% predicted or on chronic systemic steroid therapy aimed at lung disease. Data are presented as *n* (%) and continuous variables are presented as median (interquartile range)

*BSA* body surface area; *NYHA* New York Heart Association; *STS score* Society of Thoracic Surgeons score; *PCI* percutaneous coronary intervention; *CABG* coronary artery bypass grafting

Procedural and clinical outcomes are presented in Table 2a. Majority of TAVR were performed by femoral access in both groups (93.1% vs. 86.5%, *p = *0.001). In BAV group, self-expandable devices (Corevalve, Evolut R and Evolut PRO) were used more frequently than in TAV group (43.5% vs. 21.7%).

**Table 2:** Procedural and clinical outcomes (a), comparison of prosedural outcome; SAPIEN XT vs. SAPIEN3 (b) and comparison of prosedural outcome; Corevalve vs, Evolut R or Pro (c)

(a)	TAV group	BAV group	*p* value
*N*	16,802	423	
Elective procedure			
Yes	16,556 (98.5)	411 (97.2)	0.02
No	246 (1.5)	12 (2.8)	
Anesthesia			
Moderate sedation	2861(17.0)	77 (18.2)	0.53
General	13,941 (83.0)	346 (81.8)	
Approach site			
Femoral	14,542 (86.5)	394 (93.1)	< 0.001
Iliac	279 (1.7)	8 (1.9)	
Direct aorta	146 (0.9)	7 (1.7)	
Apical	1566 (9.3)	8 (1.9)	
Subclavian	238 (1.4)	6 (1.4)	
Others	31 (0.2)	0 (0.0)	
Device			
Sapien XT	4294 (25.6)	18 (4.3)	< 0.001
Sapien 3	8835 (52.6)	221 (52.2)	
Corevalve	590 (3.5)	44 (10.4)	
Evolut R	2601 (15.5)	114 (27.0)	
Evolut PRO	446 (2.7)	26 (6.1)	
No use	36 (0.2)	0 (0.0)	
Valve size			
20 mm	859 (5.1)	17 (4.0)	< 0.001
23 mm	7770 (46.2)	105 (24.8)	
26 mm	5985 (35.6)	194 (45.9)	
29 mm	2152 (12.8)	107 (25.3)	
No use	36 (0.2)	0 (0.0)	
Device success	16,127 (96.0)	404 (95.5)	0.62
Conversion to surgery	184 (1.1)	2 (0.5)	0.34
Annulus rupture	38 (0.2)	0 (0.0)	
LV rupture	46 (0.3)	1 (0.2)	
Aortic dissection	8 (0.0)	0 (0.0)	
Valve migration	10 (0.1)	0 (0.0)	
Coronary obstruction	5 (0.0)	0 (0.0)	
Others	77 (0.5)	14 (3.3)	
Post procedural AR moderate or severe	323 (1.9)	13 (3.2)	0.09
Perivalvular AR	290 (1.7)	13 (3.2)	
Transvalvular AR	15 (0.1)	0 (0.0)	
Complications within 30 days			
Major bleeding	54 (0.3)	1 (0.2)	1.00
Ischemic stroke	213 (1.3)	8 (1.9)	0.26
New pacemaker implantation	880 (5.2)	19 (4.5)	0.50
30-day mortality	214 (1.3)	2 (0.5)	0.18

(b)	Sapien XT	Sapien 3	*p* value
*N*	4312	9056	
Periprocedural outcomes			
Device success	4115 (95.4%)	8749 (96.6%)	< 0.001
Conversion to surgery	81 (1.9%)	58 (0.6%)	< 0.001
Postprocedural AR moderate or severe	87 (2.1%)	89 (1.0%)	< 0.001
30-day mortality	69 (1.6%)	79 (0.9%)	< 0.001

(c)	Corevalve	Evolut R or PRO	*p* value
*N*	634	3187	
Periprocedural outcomes			
Device success	592 (93.4%)	3069 (96.3%)	< 0.001
Conversion to surgery	20 (3.2%)	19 (0.6%)	< 0.001
Postprocedural AR moderate or severe	31 (5.1%)	126 (4.1%)	< 0.001
30-day mortality	9 (1.4%)	53 (1.7%)	< 0.001

Major bleeding includes life-threatening bleeding, bleeding that requires a transfusion of ≥ 4 units of red blood cells, bleeding associated with a hemoglobin drop of ≥ 5 g/dL, bleeding causing hypovolemic shock or severe hypotension requiring vasopressors or surgery. Data are presented as *n* (%)

Successful device deployment rate was high in both groups (95.5% vs. 96.0%, *p = *0.62), and the rate of conversion to surgery was low (0.5% vs. 1.1%, *p = *0.34). Frequency of post procedural moderate or severe aortic regurgitation was similar in both groups (3.2% vs. 2.0%, *p = *0.09). There was no statistically significant difference in frequency of complications within 30 days including major bleeding (0.2% vs. 0.3%, *p = *1.00), ischemic stroke (1.9% vs. 1.3%, *p = *0.26) and pacemaker implantation (4.5% vs. 5.2%, *p = *0.50). 30-day mortality was also similar between two groups (0.5% vs. 1.3%, *p = *0.18).

Procedural outcomes were compared between early- and new-generation devices (Table 2b, c). About balloon-expandable device, new-generation device (Sapien 3) showed better outcome than early-generation device (Sapien XT) (device success: 96.6% vs. 95.4%, *p < *0.001; conversion to surgery: 0.6% vs. 1.9%, *p < *0.001; post-procedural AR moderate to severe: 1.0% vs. 2.1%, *p < *0.001; 30-day mortality: 0.9% vs. 1.6%, *p < *0.001). As for self-expandable devices, new-generation device (Evolut series) showed also mostly better outcome than early-generation device (Corevalve) except for 30-day mortality (device success: 96.3% vs. 93.4%, *p < *0.001; conversion to surgery: 0.6% vs. 3.2%, *p < *0.001; post-procedural AR moderate to severe: 4.1% vs. 5.1%, *p < *0.001; 30-day mortality: 1.7% vs. 1.4%, *p < *0.001).

**Fig. 2:**
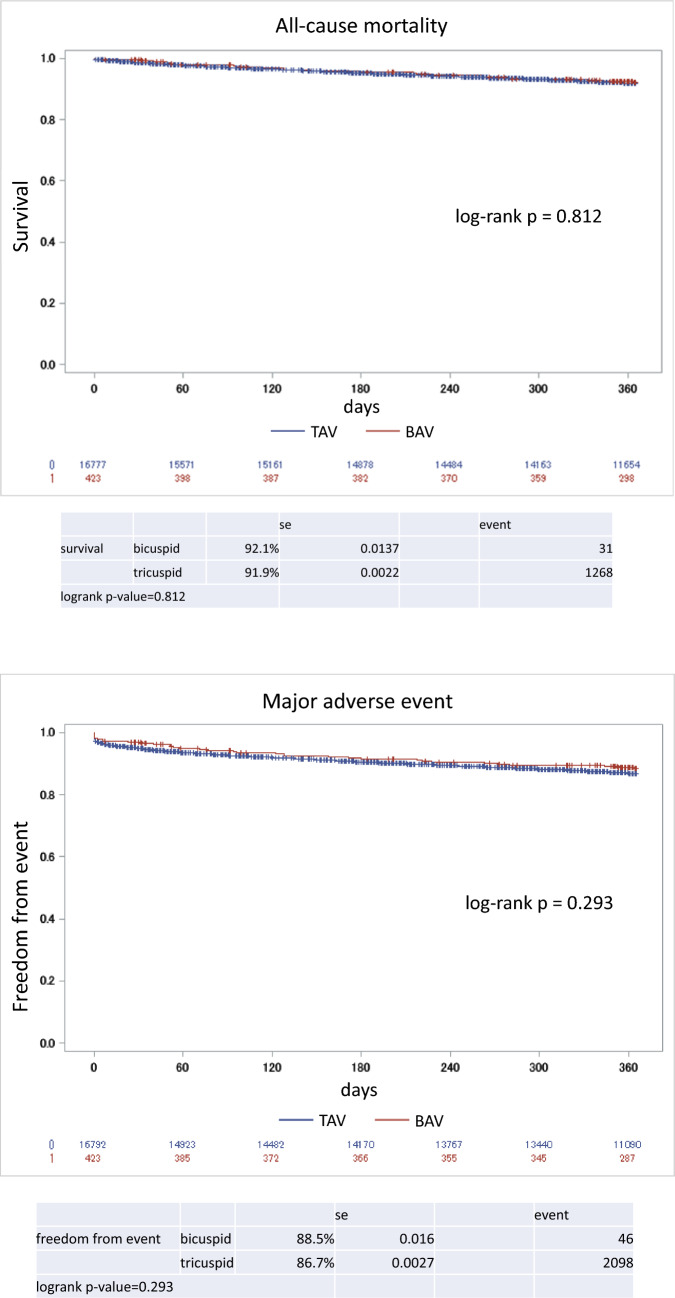
Kaplan–Meier curves for all-cause mortality and major adverse event

Kaplan–Meier survival curves for 1-year mortality and major adverse event are presented in Fig. 2. Overall survivals at 1 year were 92.1% for BAV group and 91.9% for TAV group. Similarly, survival from major adverse events were 88.5% for BAV and 86.7% for TAV. There was no significant difference in cumulative all-cause mortality and major adverse event between the groups. After the adjustments for patients’ preprocedural clinical characteristics, relative hazard for all-cause mortality for BAV compared to TAV was 1.01 (0.70–1.45; *p* value 0.96), and for major adverse event was 0.94 (0.69–1.27; *p* value 0.67) (Table 3).

**Table 3:** Cox regression analysis for the association with all-cause mortality (a) and major adverse event (b)

(a)	HR	HR lower	HR upper	Pr > ChiSq
Bicupid aortic valve	1.01	0.70	1.45	0.962
Elective	1.51	1.08	2.09	0.015
Age < 65 years	1.84	0.82	4.14	0.141
Age < 75 years	1.17	0.87	1.56	0.292
Age < 85 years	1.06	0.94	1.19	0.372
Male	0.50	0.43	0.58	< 0.0001
BSA100	0.99	0.98	0.99	< 0.0001
NYHA 3–4	1.19	1.05	1.35	0.008
STS score	1.03	1.02	1.03	< .0001
Hypertension	0.93	0.81	1.07	0.300
Hyperlipidemia	0.69	0.61	0.78	< 0.0001
Diabetes mellitus	1.16	1.02	1.32	0.022
Chronic lung disease	1.48	1.26	1.74	< 0.0001
Coronary artery disease	0.94	0.75	1.18	0.582
Non cardiac artery disease	1.39	1.20	1.61	< 0.0001
Prior PCI	1.13	0.90	1.42	0.306
Prior CABG	1.08	0.76	1.53	0.678
Prior heart surgery	1.17	0.90	1.52	0.234
Cerebrovascular disease	1.03	0.87	1.21	0.765
Pacemaker	1.00	0.80	1.25	0.994
Aortic calcification	1.25	1.05	1.49	0.012
Malignancy	1.65	1.40	1.93	< 0.0001
Hemoglobin	0.91	0.87	0.94	< 0.0001
Albumin	0.59	0.53	0.65	< 0.0001
Creatinine	1.03	1.00	1.06	0.096
Mean aortic valve gradient	0.99	0.98	0.99	< 0.0001
AV area	0.91	0.75	1.09	0.296
LVEF	1.01	1.00	1.01	0.036
Aortic insufficiency 3–4	0.95	0.78	1.17	0.631
Mitral insufficiency 3–4	1.02	0.84	1.24	0.839
Warfarin	1.26	1.04	1.53	0.021
Aspirin	0.97	0.86	1.10	0.617
Clopidogrel or prasugrel	0.93	0.80	1.07	0.289
Ticlopidine	1.30	0.71	2.35	0.396

(b)	HR	HR lower	HR upper	Pr > ChiSq
Bicupid aortic valve	0.94	0.69	1.27	0.6675
Elective	1.27	0.95	1.70	0.1011
Age < 65 years	1.83	0.95	3.55	0.0727
Age < 75 years	1.14	0.90	1.44	0.2791
Age < 85 years	1.09	0.99	1.20	0.0825
Male	0.65	0.58	0.73	< 0.0001
BSA100	0.99	0.98	0.99	< 0.0001
NYHA 3–4	1.19	1.07	1.31	0.0007
STS score	1.02	1.01	1.02	< 0.0001
Hypertension	1.10	0.98	1.23	0.0976
Hyperlipidemia	0.82	0.75	0.90	< 0.0001
Diabetes mellitus	1.11	1.00	1.23	0.0443
Chronic lung disease	1.43	1.24	1.63	< 0.0001
Coronary artery disease	0.91	0.76	1.09	0.3166
Non cardiac artery disease	1.36	1.21	1.52	< 0.0001
Prior PCI	1.22	1.01	1.46	0.0373
Prior CABG	0.96	0.73	1.27	0.7855
Prior heart surgery	1.16	0.95	1.43	0.1552
Cerebrovascular disease	1.10	0.97	1.25	0.1383
Pacemaker	0.99	0.83	1.17	0.8643
Aortic calcification	1.18	1.03	1.35	0.0201
Malignancy	1.33	1.16	1.52	< 0.0001
Hemoglobin	0.92	0.89	0.94	< 0.0001
Albumin	0.88	0.82	0.96	0.002
Creatinine	1.03	1.01	1.06	0.0135
Mean aortic valve gradient	0.99	0.99	0.99	< 0.0001
AV area	0.95	0.82	1.10	0.4755
LVEF	1.00	0.99	1.00	0.2363
Aortic insufficiency 3–4	0.93	0.79	1.10	0.3859
Mitral insufficiency 3–4	1.10	0.95	1.28	0.2008
Warfarin	1.31	1.12	1.52	0.0008
Aspirin	0.99	0.90	1.09	0.7967
Clopidogrel or prasugrel	1.00	0.89	1.11	0.9466
Ticlopidine	0.81	0.46	1.43	0.4699

## Discussion

The present study is the first large-scale study to compare the clinical outcomes of TAVR in patients with BAV and TAV in Japan. In this study, patients with stenotic BAV were treated with TAVR safely in Japan, and 1-year outcome was similar in both groups.

The largest concern regarding TAVR in BAV is aortic root injury due to the asymmetrical nature of BAV; however, we observed low frequency of annulus rupture and conversion to surgery. From the baseline characteristics, there seem to be two reasons to explain the favorable procedural outcome of TAVR in BAV. First, self-expandable devices were used more frequently in BAV group. Self-expandable devices, including Corevalve, Evolut R and Evolut PRO, are safer at aortic root rupture than balloon-expandable devices [15]. Second, Access-related complications could be limited in BAV group. BAV patients had less arteriosclerotic comorbidities, therefore, femoral access was selected more frequently in BAV group than TAV group (93.1% vs. 86.5%, *p < *0.001). Invasive alternative access represented by apical approach was rarely chosen in BAV patients. These advantages might work to cover the weak points of TAVR in BAV.

Compared to early-generation devices, new-generation devices showed mostly better procedural outcome. New-generation devices with improved functions including an external sealing cuff of Sapien 3 or retrievable system of Evolut series have advantage in paravalvular leak and optimal positioning [4]. It is expected that constantly advancing technology will make TAVR safer and more efficient regardless of valve morphology.

Implanted valve function is also major concern about TAVR in BAV. We observed no significant difference in post procedural moderate or severe aortic regurgitation in this study, but we could not investigate post-procedural valve gradients because the detail echocardiographic data was not collected in the J-TVT registry. Though the previous study reported that there was no significant difference in residual pressure gradient [4], we sometimes experience insufficient expansion of transcatheter valves implanted into severely calcified BAVs. It was reported that calcified raphe and excessive leaflet calcification were associated with increased risk of procedural and mid-term mortality [16]. Since the ellipse-shaped expansion of transcatheter valves may affect their functions [17], long-term follow-up with echocardiographic examination will be needed.

Supported by favorable outcomes of TAVR for lower-risk patients [18, 19], the indication of TAVR in TAV is now expanding to younger and lower surgical risk patients. This trend will lead to increased proportion of BAV patients in the candidates for TAVR. When we treat younger and lower-risk patients, it is even more important to consider their long-term morbidity and mortality. Further investigation about long-term valve durability and incidence of aortic complications will be necessary to demonstrate the feasibility of TAVR in BAV patients.

## Study limitations

There are several limitations in the present study. First, we did not have a core laboratory of echocardiography. The grading criteria of PVL was determined in each institution. Second, Anatomical measurements of contrast CT, such as the coronary height, the size of sinus of Valsalva and severity of calcification, are also analyzed in each institution, not in a core laboratory. There is no information about BAV phenotypes. These data may influence the optimal selection of device type and device size. Third, the details of each procedure were not considered in this study. For example, balloon expandable devices are often implanted into severely calcified aortic valve with under-filling technique to avoid aortic root injury. Fourth, the operator experiences were not considered in this study. Finally, 1-year echocardiographic outcome was not available. Further long-term investigation along with echocardiographic examination are needed to evaluate the efficacy of TAVR in BAV patients.

## Conclusions

From the Japanese national database of TAVR, procedural and 1-year outcome of TAVR in BAV was comparable to TAVR in TAV. Further investigation about long-term valve function and outcome will be needed.

## Data Availability

The datasets generated and/or analyzed during the current study are not publicly available due to regulatory restrictions imposed by Japan Transcatheter Valve Therapies (JTVT). However, interested parties may request access to the data from the corresponding author, subject to reasonable request and authorization.

## References

[1] Hoffman JI, Kaplan S (2002). The incidence of congenital heart disease. J Am Coll Cardiol.

[2] Yeats BB, Yadav PK, Dasi LP, Thourani VH (2022). Treatment of bicuspid aortic valve stenosis with TAVR: filling knowledge gaps towards reducing complications. Curr Cardiol Rep.

[3] Gollmann-Tepekoylu C, Nagele F, Engler C, Stoessel L, Zellmer B, Graber M (2022). Different calcification patterns of tricuspid and bicuspid aortic valves and their clinical impact. Interact Cardiovasc Thorac Surg.

[4] Yoon SH, Bleiziffer S, De Backer O, Delgado V, Arai T, Ziegelmueller J (2017). Outcomes in transcatheter aortic valve replacement for bicuspid versus tricuspid aortic valve stenosis. J Am Coll Cardiol.

[5] Waksman R, Craig PE, Torguson R, Asch FM, Weissman G, Ruiz D (2020). Transcatheter aortic valve replacement in low-risk patients with symptomatic severe bicuspid aortic valve stenosis. JACC Cardiovasc Interv.

[6] Forrest JK, Ramlawi B, Deeb GM, Zahr F, Song HK, Kleiman NS (2021). Transcatheter aortic valve replacement in low-risk patients with bicuspid aortic valve stenosis. JAMA Cardiol.

[7] Kong WKF, Regeer MV, Poh KK, Yip JW, van Rosendael PJ, Yeo TC (2018). Inter-ethnic differences in valve morphology, valvular dysfunction, and aortopathy between Asian and European patients with bicuspid aortic valve. Eur Heart J.

[8] Watanabe Y, Hayashida K, Takayama M, Mitsudo K, Nanto S, Takanashi S (2015). First direct comparison of clinical outcomes between European and Asian cohorts in transcatheter aortic valve implantation: the Massy study group vs. the PREVAIL JAPAN trial. J Cardiol.

[9] Handa N, Kumamaru H, Torikai K, Kohsaka S, Takayama M, Kobayashi J (2018). Learning curve for transcatheter aortic valve implantation under a controlled introduction system- initial analysis of a Japanese Nationwide registry. Circ J.

[10] Genereux P, Piazza N, Alu MC, Nazif T, Hahn RT, Pibarot P (2021). Valve Academic Research Consortium 3: updated endpoint definitions for aortic valve clinical research. Eur Heart J.

[11] Yoon SH, Ahn JM, Hayashida K, Watanabe Y, Shirai S, Kao HL (2016). Clinical outcomes following transcatheter aortic valve replacement in Asian population. JACC Cardiovasc Interv.

[12] Mack MJ, Brennan JM, Brindis R, Carroll J, Edwards F, Grover F (2013). Outcomes following transcatheter aortic valve replacement in the United States. JAMA.

[13] Forrest JK, Kaple RK, Ramlawi B, Gleason TG, Meduri CU, Yakubov SJ (2020). Transcatheter aortic valve replacement in bicuspid versus tricuspid aortic valves from the STS/ACC TVT registry. JACC Cardiovasc Interv.

[14] Makkar RR, Yoon SH, Leon MB, Chakravarty T, Rinaldi M, Shah PB (2019). Association between transcatheter aortic valve replacement for bicuspid vs tricuspid aortic stenosis and mortality or stroke. JAMA.

[15] Sa M, Simonato M, Van den Eynde J, Cavalcanti LRP, Alsagheir A, Tzani A (2021). Balloon versus self-expandable transcatheter aortic valve implantation for bicuspid aortic valve stenosis: a meta-analysis of observational studies. Catheter Cardiovasc Interv.

[16] Yoon SH, Kim WK, Dhoble A, Milhorini Pio S, Babaliaros V, Jilaihawi H (2020). Bicuspid aortic valve morphology and outcomes after transcatheter aortic valve replacement. J Am Coll Cardiol.

[17] Zegdi R, Ciobotaru V, Noghin M, Sleilaty G, Lafont A, Latremouille C (2008). Is it reasonable to treat all calcified stenotic aortic valves with a valved stent? Results from a human anatomic study in adults. J Am Coll Cardiol.

[18] Mangieri A, Tchetche D, Kim WK, Pagnesi M, Sinning JM, Landes U (2020). Balloon versus self-expandable valve for the treatment of bicuspid aortic valve stenosis: insights from the BEAT International Collaborative registrys. Circu Cardiovasc Interv.

[19] Popma JJ, Deeb GM, Yakubov SJ, Mumtaz M, Gada H, O'Hair D (2019). Transcatheter aortic-valve replacement with a self-expanding valve in low-risk patients. N Engl J Med.

